# Effect of Schedule-Induced Behavior on Responses of Spontaneously Hypertensive and Wistar–Kyoto Rats in a Delay-Discounting Task: A Preliminary Report

**DOI:** 10.3389/fnbeh.2019.00255

**Published:** 2019-11-13

**Authors:** Sergio Ramos, Gabriela E. López-Tolsa, Espen A. Sjoberg, Ricardo Pellón

**Affiliations:** ^1^Animal Learning and Behavior Laboratory, Departamento de Psicología Básica I, Universidad Nacional de Educación a Distancia (UNED), Madrid, Spain; ^2^Animal Behavior Laboratories, Department of Behavioral Science, Oslo Metropolitan University, Oslo, Norway; ^3^Schools of Health Sciences, Kristiania University College, Oslo, Norway

**Keywords:** delay discounting, schedule-induced behavior, impulsivity, compulsivity, SHR vs. WKY rats

## Abstract

Delay discounting is the loss of the subjective value of an outcome as the time to its delivery increases. It has been suggested that organisms can become more tolerant of this delay when engaging in schedule-induced behaviors. Schedule-induced behaviors are those that develop at a high rate during intermittent reinforcement schedules without the need of arranged contingency to the reinforcer, and they have been considered as a model of compulsivity. There is evidence that relates compulsivity to greater delay discounting. The rate of delay discounting represents how impulsive the subject is, as the rate of discounting increases the higher the impulsivity. Thus, the main purpose of this study was to undertake a preliminary evaluation of whether developing schedule-induced behaviors affects performance in a delay-discounting task, by comparing spontaneously hypertensive rats (SHRs) and Wistar–Kyoto (WKY) rats. The rats were exposed to a task that consisted of presenting the subjects with two levers: one produced a small, immediate food reinforcer while the other one produced a larger, delayed reinforcer. During Condition A, the levers were presented, and a water bottle and a running wheel were available in the conditioning chambers; during Condition B, only the levers were presented. SHR and WKY rats developed schedule-induced behaviors during Condition A and showed no difference in discounting rates, contradicting previous reports. Lick allocation during response-reinforcer delays and the inter-trial interval (ITI) showed, respectively, pre- and post-food distributions. Discounting rates during Condition B (when rats could not engage in schedule-induced behaviors) did not reach statistical significance difference among strains of animals, although it was observed a tendency for WKY to behave more self-controlled. Likewise it was not found any effect of schedule-induced behavior on discounting rates, however, a tendency for WKY rats to behave more impulsive during access to drink and run seems to tentatively support the idea of schedule-induced behavior as a model of compulsivity in those rats, being impulsivity simply defined as an excess in behavior.

## Introduction

Impulsive behavior is seen in everyday life, but the nature of this heterogeneous concept means that there is no consensus on its definition. In clinical psychology, impulsivity has been defined as a wide range of symptoms or factors that interact with each other, including impatience, interruption of activities, difficulty to plan ahead, difficulty to wait, excessive spending and/or substance abuse (American Psychiatric Association, 2013). On the other hand, impulsivity has been operationally defined in animal behavior research as a preference for smaller immediate reinforcers over large delayed ones (Fox et al., 2008; Hamilton et al., 2015), as an aversion to the delay (Sonuga-Barke et al., 1992; Richards et al., 2011), or as the inability to wait or to withhold a response (Richards et al., 2011). Although the operational definition of impulsivity seems to be heterogeneous as well, Sosa and dos Santos (2019) argue that different paradigms study the same behavioral tendency.

Impulsivity has been studied using different standardized intertemporal choice procedures, like the simple- and adjusting-delay discounting tasks (Sosa and dos Santos, 2019). One of the advantages of using standardized procedures is that they facilitate the comparison of analogous behaviors across species (Richards et al., 2011), although in some cases standardization is reduced due to methodological changes associated with accommodating the procedures to various species (Sjoberg, 2017).

Delay discounting is the loss of the subjective value of an outcome as the time to its delivery increases (Vanderveldt et al., 2016). Impulsivity has been evaluated using delay-discounting tasks since Mazur (1987) proposed the use of an adjusting-delay procedure (Sjoberg and Johansen, 2018; see also Bickel et al., 2015). In Mazur’s experiment, pigeons were presented with a choice between two options, one that gave a small reward after a short delay, and the other that gave a large reward after a long delay. The value of the longer delay increased or decreased across the blocks of trials depending on the subject’s responses, while the delay to the small reward was held constant. The purpose of this adjusting-delay procedure was to find out the subjective indifference point at which the value of both options was the same for each given subject (Vanderveldt et al., 2016).

One variation of the procedure suggested by Mazur (1987) is the simple delay-discounting procedure, which consists on presenting the subject with two options: one that gives a small immediate reinforcer (SS) and another that gives a larger delayed reinforcer (LL; Fox et al., 2008). When using animals, the delay to LL increases throughout the experiment and the animals usually start preferring the LL option until the delay reaches a value at which organisms change their preference to the SS option (Fox et al., 2008). Contrary to the adjusting-delay procedure, the value of the delay changes independently of the subject’s responses, but the time at which the subject changes its preference (choosing LL in 50% of the trials) is equivalent to the indifference point in the adjusting-delay procedure (Hamilton et al., 2015). As pointed out by Sjoberg and Johansen (2018), this means that discounting behavior could be interpreted in one of two ways: either as a continuous measure, showing a degree of impulsive behavior, or as a switch in preference from large to small reinforcers. The more sensitive the subject is to the delay, the more impulsive it will be considered. Mazur (1987) also proposed a hyperbolic function as a mathematical model to describe the discounting rate, which predicts the preference reversal in humans and in different animal species, suggesting that different organisms discount in a similar way (Vanderveldt et al., 2016).

Two different strains of rats have been typically used to study impulsivity using delay-discounting tasks: the Spontaneously Hypertensive Rat (SHR) and the Wistar–Kyoto (WKY) rat (Adriani et al., 2003; Fox et al., 2008; Hand et al., 2009; Aparicio et al., 2019). The SHR is considered a valid rodent model of Attention-Deficit/Hyperactivity Disorder (ADHD) because rats from this strain exhibit behavioral characteristics similar to those seen in humans with ADHD, such as impaired sustained attention, learning insufficiencies, resistance to extinction, hyperactivity, hypersensitivity to delayed consequences, impulsivity, motor impulsiveness and behavioral variability (Sagvolden, 2000; Fox et al., 2008; Sontag et al., 2010; Orduña, 2015; Aparicio et al., 2019). WKY rats, on the other hand, do not exhibit so much excessive behavior and they are more tolerant of delayed consequences, so this strain is commonly used as a control group for the SHR. Its validity as a control has been questioned, as differences exist across vendor strains, with the WKY/NHsd strain being proposed as a model of inattention (Sagvolden et al., 2008, 2009; Sagvolden and Johansen, 2012). Other strains of rats, such as Lewis and Fisher 344 (Anderson and Woolverton, 2005) or Roman High- and Low-Avoidance (Moreno et al., 2010), have been also used to study impulsivity, although SHR and WKY rats have been the most used (Sontag et al., 2010).

Fox et al. (2008) carried out an experiment to determine if SHR behaved more impulsively than WKY rats. For that, they used a delay-discounting task in which two levers were presented: a press to one of the levers delivered one food pellet immediately, while a press to the other delivered three food pellets after a delay. This delay increased as the experiment progressed, and subsequently decreased. SHRs chose the LL option significantly less often than WKY rats, regardless of delay order. SHRs will also express higher degrees of impulsivity if the delays are presented in random order (Fox et al., 2008; Aparicio et al., 2019).

The delay-aversion theory proposes that the delay function should be understood as the overall trial length or the overall waiting time, meaning that impulsivity is the result of an inability to endure long trials in order to secure large rewards (Sonuga-Barke et al., 1992). However, as reviewed by Sjoberg and Johansen (2018), this theory holds little validity in animal research. Instead, the delay between response and reinforcer is the strongest predictor of impulsivity in animal research, although in humans both the delay and the trial length together explain the phenomenon, known as the dual-component model (Marco et al., 2009).

It was recently suggested that schedule-induced behavior might help organisms improve their performance in temporal tasks such as the temporal bisection or fixed-interval schedules (Ruiz et al., 2016), differential reinforcement of low rates (DRL; Bruner and Revusky, 1961; Segal and Holloway, 1963), or the peak procedure (Mattel and Portugal, 2007), by providing an alternative activity for the organisms. Seemingly, schedule-induced behaviors can make waiting-time less aversive, so having the opportunity to engage in them should make organisms more self-controlled. Furthermore, DRL schedules and the peak procedure have been considered tasks that also measure impulsivity (see Pellón et al., 2018), although they were not designed to study choice (Mattel and Portugal, 2007; Sosa and dos Santos, 2019). Better performance on these tasks would imply that subjects are behaving more self-controlled.

When organisms are trained under intermittent reinforcement schedules they normally develop excessive patterns of behaviors during the inter-reinforcers intervals, even when there is no explicit contingency between their occurrence and the delivery of the reinforcer; those behaviors are called schedule-induced behaviors (Falk, 1971; Killeen and Pellón, 2013). Traditionally, schedule-induced behaviors have been included in a category different than operants (Falk, 1971) because they seem to be induced by a low probability of reinforcement (Staddon, 1977). Nevertheless, Pellón et al. (2018) have recently proposed that schedule-induced behaviors are induced by events in the environment and maintained by delayed reinforcement (Killeen and Pellón, 2013; Ruiz et al., 2016; Álvarez et al., 2016).

The most studied example of schedule-induced behavior is schedule-induced drinking, which consists of a small, regular and persistent drinking after each food pellet is delivered when food-deprived rats are exposed to an intermittent food-presentation schedule (Íbias and Pellón, 2011). After some training, the high pattern of drinking concentrates in the first 15–20 s of the interval (Álvarez et al., 2016), although if access to water is restricted to the last part of the interval, it will develop showing a similar distribution in latter portions of the interval (López-Crespo et al., 2004).

On the other hand, it has been suggested that schedule-induced drinking is a model of compulsivity (Moreno and Flores, 2012). Compulsivity is defined as performing an act persistently and repetitively, inappropriate to the situation, and with no obvious relation to the overall goal, in order to prevent perceived negative consequences leading to functional impairment of the organism (Oldham et al., 1996; Dalley et al., 2011). Considering the definitions of compulsion and schedule-induced behavior, it can be noticed that both occur persistently, repetitively and with no obvious relationship to the overall goal (there is no arranged contingency between the behavior and the obtaining of reinforcement, though relations can be established based on the notion of proximity—see Killeen and Pellón, 2013).

It has been observed that patients with Parkinson’s disease and Obsessive-Compulsive Disorder (OCD) with comorbid impulsive-compulsive behaviors showed elevated delay-discounting rates compared to patients without comorbid compulsive behavior or healthy participants (Housden et al., 2010; Pinto et al., 2014; Sohn et al., 2014). If schedule-induced behavior functions as compulsive behavior, its development should make other organisms behave more impulsive, as it has been observed with humans. SHR rats could serve as a model of patients with comorbid impulsive-compulsive behaviors because of their elevated discounting rate and excessive behavior compared to WKY rats that could simulate the discounting rates of the patients that do not develop a comorbid compulsive behavior.

The aim of the present study was to observe the effect of having the opportunity to develop schedule-induced behavior during a delay-discounting task. To achieve that, rats were exposed to a delay-discounting task and the experiment was run in two successive conditions, one in which subjects could develop schedule-induced behavior, and the other in which they could not. If schedule-induced behavior acts as compulsive behavior, rats will show steeper discounting (higher degree of impulsivity) when the opportunity to develop schedule-induced behavior is available. By contrast, if schedule-induced behavior causes waiting time to be less aversive rats under schedule-induced behavior should discount less, i.e., be less impulsive.

## Materials and Methods

### Subjects

Six SHR rats and six WKY rats were used as subjects in this experiment. SHRs were obtained from Janvier Laboratories (France) and WKY rats from Envigo Laboratories (United Kingdom). The different origin of the animals is based on findings that support differences across vendors in the responsiveness of specific strains of rats (Sagvolden et al., 2009). The average group weight at the beginning of the experiment was 245.3 g (range: 225–278) for SHR and 237.5 g (range: 210–264) for WKY. At the beginning of the experiment, all subjects were 15 weeks old. They were housed individually in an environmentally controlled room where temperature was 22°C, relative humidity was maintained at 55%, and there was a 12:12 h light-dark cycle (lights on at 8:00 AM). The home cages were made of transparent Plexiglas and measured 18 × 32.5 × 20.5 cm. Experimental sessions were conducted during the light part of the cycle, Monday through Sunday, at about the same time every day. Subjects were maintained at 85% of their free-feeding weights, following the standard growth curve for each strain, by restricting the amount of food they received every day. Water was freely available in the home cages. Each rat was weighed daily before the experimental session and supplemental feeding was delivered between 30 min and 1 h after the experimental session ended. All rats only had previous testing experience with the same delay-discounting task using both levers of the conditioning chambers, but they had no previous experience with schedule-induced behavior or any other experimental preparation.

### Apparatus

Sessions were conducted using eight Letica LI-836 conditioning chambers. The conditioning chambers measured 29 × 24.5 × 35.5 cm and were enclosed in sound-attenuating boxes with a fan mounted on one of the walls that provided an ambient noise of approximately 60 dB. The front wall of each chamber was made of aluminum, the right and the rear walls were made of black Plexiglas, and the remaining wall was made of transparent Plexiglas. The floor consisted of a 16-bars stainless metal grid. The front panel of each chamber was equipped with two levers located at each side and a food tray located between them, 3.7 cm above the floor. The right wall had a 3.2 cm × 3.9 cm aperture situated 20 cm from the front panel and 7 cm above the floor. A bottle of water could be mounted behind the wall, and the rat could reach the spout from inside the aperture. Licks were recorded through the contact of the rat’s tongue to the spout, which completed an electric circuit between the floor and the spout. At the rear wall, access to an activity wheel mounted outside the conditioning chambers was permitted. The activity wheel was made of stainless metal, measured 9 cm wide and had a diameter of 34 cm. Turns in the wheel were recorded using a magnet system which counted a turn each time it was closed. The houselight was mounted behind the front panel and provided general illumination during the sessions. Forty-five miligram food pellets (Bio-Serv, Frenchtown, NJ, USA) were delivered into the food tray. A MED-PC application under a Microsoft Windows XP environment provided environmental control and recorded lever presses, licks, and turns.

### Procedure

The present experiment consisted of a delay-discounting task, in which two levers were presented to the subjects; after a response, one lever delivered one food pellet immediately and the other delivered three food pellets after an increasing delay. The experiment followed an A-B design, so each subject faced the task twice, once in each condition. Each condition began with some pre-training (see below).

During the delay-discounting phase in Condition A, rats had access to the bottle with water and the running wheel in the conditioning chambers. Access to the bottle and the wheel was not permitted in Condition B. Each bottle was filled with 150 ml of fresh tap water before experimental sessions.

Sessions in all phases were divided into 10 blocks. Each block consisted of six trials: two forced and four free trials. Trials started when the levers were inserted into the chamber after a response the levers were retracted, the food pellet(s) delivered and a 10-s inter-trial interval (ITI) began (except during the delay-discounting phase, more details provided in a section below). During forced trials only one lever was presented at a time, the order of which was randomized, and during free trials, both levers were presented. The houselight was turned on at the beginning of the experimental sessions and turned off at the end.

#### Pre-training

During phase 1 of pre-training, the two levers delivered one food pellet immediately after a single response (FR1). The aim of this phase was to control for lever-bias, such that if a rat showed a preference for one lever over the other, with all else being equal, then this could be counter-balanced when assigning SS and LL levers. Assigning the large reinforcement to the less-preferred lever ensured that rats would press it because of the magnitude of the reinforcer, not because of lever-bias. This phase lasted one session.

Phase 2 was similar to the prior one, except that one of the levers delivered three food pellets and the other delivered just one. The lever that delivered three food pellets was the opposite of the preferred one during the previous phase. The purpose of phase 2 was to conduct a preference test, ensuring that rats preferred a large reinforcer (three food pellets) rather than a small reinforcer (one food pellet) in the absence of any experimental manipulation. Rats stayed in this phase until they chose the larger option in at least 66% of the trials during three consecutive sessions. Rats that did not reach the criterion in a maximum of 10 sessions were removed from the experiment.

**Figure 1:**
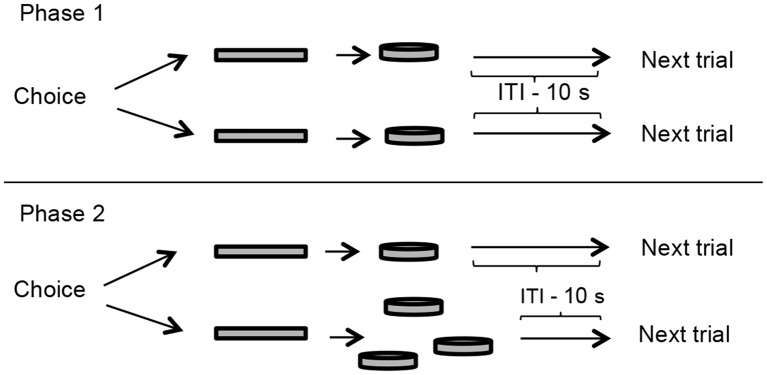
Pre-training outline. In phase 1 (upper panel), two levers (represented by rectangles in this diagram) were presented to the rats, both delivered one food pellet immediately after a press, but only one could be pressed in every trial. The goal of this phase was to measure the lever-bias of each rat, and it lasted one session. Phase 2 (lower panel) was a preference test for the large reinforcer. One of the levers delivered one food pellet and the other delivered three food pellets, both immediately. This phase lasted 10 sessions or until the subject reached the criterion of 66% of the choices to the large reinforcer in three consecutive sessions. Presentation of food pellets was followed by a 10-s inter-trial interval (ITI) in which levers were retracted.

A schematic representation of the pre-training is outlined in Figure 1.

#### Delay Discounting

**Figure 2:**
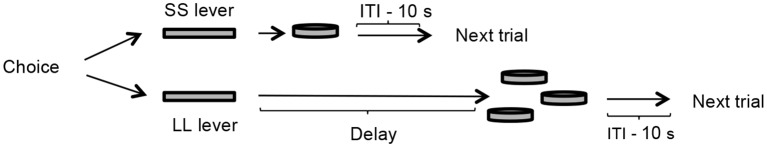
The delay-discounting task consisted of presenting the rat two options: one that delivered one food pellet immediately (SS lever), and the other that delivered three food pellets after a delay (LL lever). After the delivery of the food pellets in both options, a 10-s ITI started, and then a new trial began. The value of the delay increased 3 s per session until a maximum of 36 s, thus during each individual session the value of the delay remained constant.

This phase consisted of a delay-discounting task, during which two levers were presented to the rats: a press to one of the levers delivered one food pellet immediately (SS option) and one press to the other lever delivered three food pellets after a delay (LL option). Trials were similar to previous phases, except that after a press to the LL lever, the lever was retracted, and the delay started, the three food pellets were delivered after the delay, and then the 10-s ITI began. Therefore, the trial length grew as the value of the delay increased for the LL option. Because sessions finished after 60 choices regardless of the trials’ duration, it was controlled the potential influence of reinforcement rate on choice. The delay between the response and the reinforcer increased 3 s per session until 36 s. So, the delays were: 0, 1, 3, 6, 9, 12, 15, 18, 21, 24, 27, 30, 33 and 36 s. A 1-s delay was included to illustrate the difference between the presence and absence of a delay, even if the delay was short. The delay-discounting phase lasted 14 sessions. See Figure 2 for a schematic representation of the task.

### Data Analysis

Lever presses, licks and wheel turns were recorded. The percentage (%) of responses to the LL option in the free choice trials was calculated considering data from the free choice trials only. The indifference point is 50%, so if the percentage of LL responses was above 50%, subjects were considered to be behaving self-controlled, while an LL percentage below 50% is indicative of impulsive behavior. The number of licks and turns were recorded every 1-s bin. The mean number of licks for each session and subject were calculated, as well as the mean number of licks per 1-s bin during the response-reinforcer delays and the ITI. The average of licks during each 1-s bin was calculated by dividing the number of licks in each bin by the times that bin occurred during the experiment. For example, the mean licks in bin 1 included data from all the delays, whereas the mean licks in bin 30 included data from delays of 30 s and longer.

Differences in the percentage of LL responses were analyzed using an analysis of variance (ANOVA) with a repeated measures factor, delay value (with 14 levels), and fixed factors: strain (with two levels), and condition (with two levels). Differences in the proportion of licks were analyzed using an ANOVA with a repeated measures factor, bins (with 36 levels for the proportion of licks during the delay, and 10 levels for the proportion of licks during the ITI), and fixed factors: strain (with two levels), and ITI (with two levels). Differences between strains in the total licks per session were also analyzed using an ANOVA with the repeated measures factor of delay value (with 14 levels) and strain as a fixed factor (with two levels). In all cases, statistical significance was set at a minimum *p* < 0.05.

In order to provide a quantitative measure of impulsivity, data of each subject was fitted to Mazur’s Hyperbolic Model (Mazur, 1987), and the sensitivity to the delay was calculated with the following equation:

V=A/(1+kD),

where *V* represents the subjective value of the large delayed reward, *A* corresponds to the mean proportion of LL responses under 0 s delay as the curve start point, *k* is a free parameter that represents the rate of discounting, and *D* is the value of the delay. The best-fitting parameters were obtained by the least-squares method using Microsoft Excel solver, with the constraint that *k* should be greater or equal to zero (*k >* = 0). The goodness of fit was calculated using the coefficient of determination (*R*^2^). The hyperbolic function describes the hyperbolic discounting rate of the reward as the value of the delay changes. Greater values of *k* mean more impulsivity. For a better understanding of Mazur’s Hyperbolic Model, see Mazur (1987). To compare the values of *k* one-way ANOVAs were used, comparing groups (SHR and WKY) or conditions (Condition A and B) as factors. The significance level was established at a minimum of *p* < 0.05.

## Results

### Pre-training

During pre-training, a preference for the large option was acquired by all rats. At the end of pre-training of Condition A, the mean choice of the large option for SHR rats was 0.93 ± 0.02 (mean ± SEM) and for WKY rats was 0.93 ± 0.03, and at the end of pre-training of Condition B, the mean choice of the LL option for the SHR group was 0.89 ± 0.02 and 0.98 ± 0.08 for the WKY group. These results ensured that subjects chose the lever because of the magnitude of the reinforcement and not because of any side-bias for the lever.

### Temporal Discounting

**Figure 3:**
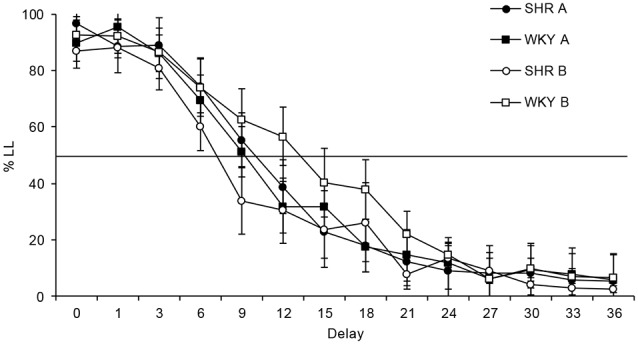
Mean percentage of responses to the LL option in each delay. Black symbols depict data from Condition A, and white symbols from Condition B. Circles represent data from spontaneously hypertensive rats (SHRs) and squares from WKY. Vertical bars denote standard error of the mean.

Figure 3 shows the mean percentage of choices for the LL lever across the delays of each strain at each condition. Schedule-induced behavior could be developed during Condition A, but not in Condition B. In both conditions a main effect was found for delay (Condition A: *F*_(3,32)_ = 148.112, *p* < 0.001, η = 0.937; Condition B: *F*_(2,22)_ = 71.415, *p* < 0.001, η = 0.877), as the duration of the delay increased, the percentage of responses to the LL option decreased. Statistical analysis of the percentage of LL responses found that differences between strains were neither significant in Condition A (*F*_(1,10)_ = 0.006, *p* = 0.940, η = 0.001), nor in Condition B (*F*_(1,10)_ = 1.517, *p* = 0.246, η = 0.132). It was also found that differences between conditions were not statistically significant for SHR (*F*_(1,10)_ = 0.491, *p* = 0.499, η = 0.047) or WKY (*F*_(1,10)_ = 0.844, *p* = 0.380, η = 0.078). Although no statistical differences were obtained, it can be observed that SHR tended to behave slightly less impulsively during Condition A in comparison to Condition B, while WKY rats behaved more impulsively during Condition A.

Table 1 shows individual values of parameter *k*, and the mean of each group in each condition. Regarding the group data in Condition A, the discounting rate represented by *k* was similar for both strains (0.15 for SHR rats, and 0.14 for WKY rats). In Condition B, however, the SHR group discounted faster (0.18) than the WKY group (0.11), but this difference was not statistically significant (*F*_(1,11)_ = 3.132, *p* = 0.107). Comparing each strain with itself in both conditions, *k* value for SHR group in Condition A (0.15) was smaller than in Condition B (0.18). Conversely, WKY rats presented a larger *k* value in Condition A (0.14) than in Condition B (0.11). Statistical comparisons between these *k* values yielded non-significant results (*p* > 0.05). These results confirm what was observed in Figure 1. No statistical differences between groups were found in the adjustment of the individual data to the model, neither between conditions nor strains (A, SHR vs. WKY: *F*_(1,11)_ = 0.161, *p* = 0.697; B, SHR vs. WKY: *F*_(1,11)_ = 0.28, *p* = 0.871; SHR, condition A vs. B: *F*_(1,11)_ = 0.403, *p* = 0.540; WKY, condition A vs. B: *F*_(1,11)_ = 0.355, *p* = 0.564).

**Table 1:** Individual and mean *k* and *R*^2^ in both conditions.

	*k*	*R*^2^		*k*	*R*^2^
	Condition A				
SHR			WKY		
1	0.11	0.82	1	0.07	0.72
2	0.24	0.87	2	0.23	0.88
3	0.18	0.95	3	0.07	0.76
4	0.14	0.79	4	0.13	0.87
5	0.09	0.78	5	0.14	0.84
6	0.14	0.81	6	0.18	0.86
Mean	0.15 ± 0.02	0.87		0.14 ± 0.02	0.87
	Condition B				
SHR			WKY		
1	0.05	0.62	1	0.05	0.67
2	0.18	0.80	2	0.18	0.88
3	0.25	0.87	3	0.16	0.89
4	0.17	0.73	4	0.05	0.69
5	0.28	0.91	5	0.10	0.78
6	0.15	0.89	6	0.12	0.85
Mean	0.18 ± 0.03	0.89		0.11 ± 0.02	0.85

*Note. Mean ± SEM. *R*^2^ is the coefficient of determination*.

### Schedule-Induced Drinking

Regarding schedule-induced behaviors, only schedule-induced drinking was developed, not schedule-induced running. Rats ran no more than 10 turns per session in at least half of the sessions during the experiment (except subjects WKY-1 and SHR-6), so data on schedule-induced running were not analyzed.

**Figure 4:**
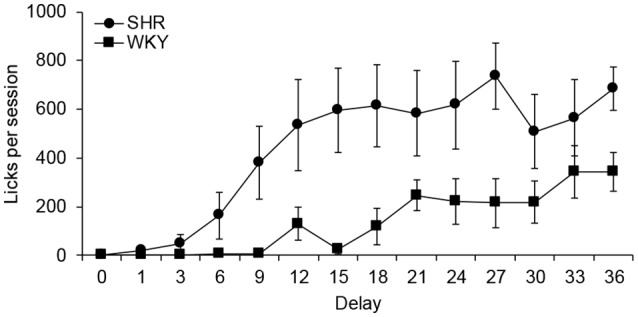
Mean total licks per session at each delay for each group. Circles depict data from SHR rats, and squares from WKY rats. Vertical bars denote standard error of the mean.

Figure 4 depicts the mean number of licks at each delay value. A main effect was obtained for strain (*F*_(1,10)_ = 6.075, *p* = 0.033, η = 0.378), with SHR giving more licks than WKY; and for delay (*F*_(3,26)_ = 15.320, *p* > 0.000, η = 0.605), with more licks being given as the delay value increased. The interaction delay × strain was also statistically significant (*F*_(3,26)_ = 3.875, *p* > 0.024, η = 0.279) and *post hoc* tests indicated that differences occurred in sessions with delay values of 9, 15, 18, 27 and 36 s. It can be discerned that SHR started developing schedule-induced drinking from the fourth session (6-s delay), showing its maximum number of licks when the delay was 27 s, with a mean of 737 licks. In contrast, WKY rats started to develop schedule-induced drinking when the delay value was 12 s, reaching the maximum number of licks when the delay was 33 s, with a mean of 342 licks. SHRs drank more than WKY rats throughout the procedure.

**Figure 5:**
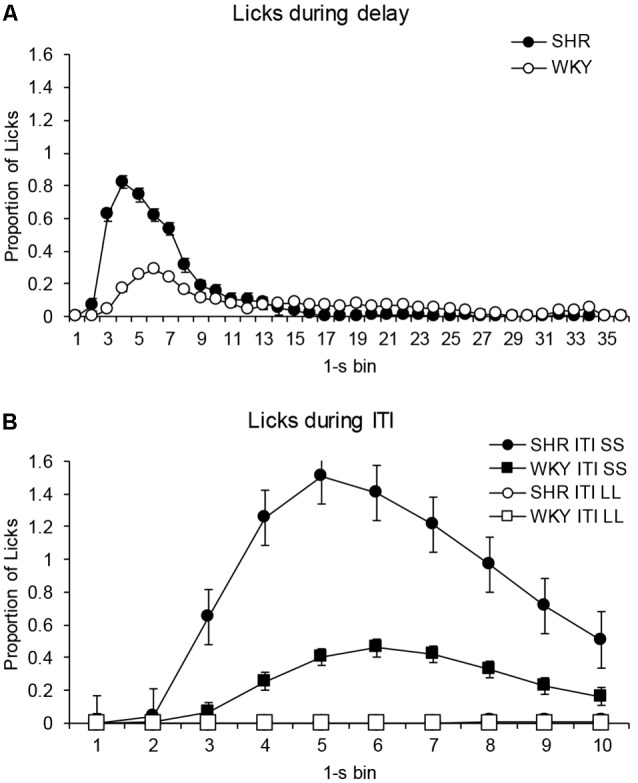
Proportion of licks for each of the 1-s bins that comprised the time intervals of the delay-discounting procedure. **(A)** Proportion of licks during the delay. Black circles depict data from SHR rats and white circles from WKY rats. **(B)** Proportion of licks during ITI. Black symbols depict data from licks given during the ITI after choosing the SS alternative, and white symbols data from the ITI after choosing the LL alternative. Circles represent data from SHR and squares from WKY. Vertical bars denote standard error of the mean.

Figure 5 shows the proportion of licks given every 1-s bin during the delay and the ITI for the average of all delay-discounting sessions. Figure 5A compares the proportion of licks in every 1-s bin during the delay for both strains of rats. The analysis revealed a main effect for bin (*F*_(2,18)_ = 9.262, *p* < 0.002, η = 0.481) and for the interaction bin × strain (*F*_(2,18)_ = 4.465, *p* < 0.031, η = 0.309), but not a main effect of strain (*F*_(1,10)_ = 2.229, *p* = 0.166, η = 0.182). *Post hoc* tests (Bonferroni) indicated that differences in favor of higher licking by SHRs occurred at bins 3 (*p* = 0.05), and 4, 5, 6 and 7 (*p* < 0.05), where licks peaked for both strains (although a bit earlier for SHR than WKY).

Figure 5B depicts the proportion of licks during the ITI after choosing LL or SS alternatives. In the ITI after choosing the LL alternative differences between strains were not significant (*F*_(1,10)_ = 2.424, *p* = 0.151, η = 0.195), neither strain licked during those time periods. However, in the ITI after choosing the SS alternative, a main effect was found for strain (*F*_(1,10)_ = 6.045, *p* < 0.034, η = 0.377), with SHR licking more than WKY rats. *Post hoc* tests (Bonferroni) indicated that differences in favor of higher licking by SHRs occurred at bins 2, 3, 4, 5, 9 and 10 (*p* < 0.05). Statistical analyses also confirmed that rats of both strains drank significantly more during the ITI after choosing the SS alternative than after choosing the LL alternative (SHR: *F*_(1,10)_ = 8.710, *p* < 0.015, η = 0.466; WKY: *F*_(1,10)_ = 6.910, *p* < 0.025, η = 0.409).

## Discussion

The purpose of the present experiment was to evaluate if schedule-induced behaviors turn waiting-time less aversive, thus making rats behave more self-controlled, or if schedule-induced behaviors could serve as a model of compulsivity, by making rats behave more impulsively. We failed to find any effect of schedule-induced behavior on delay discounting in either strain. Differences between strains are typically observed when schedule-induced behavior could not be expressed (Fox et al., 2008; Íbias and Pellón, 2011; Aparicio et al., 2019). However, some studies found no strain differences (Adriani et al., 2003; Pardey et al., 2009; Garcia and Kirkpatrick, 2013; Botanas et al., 2016), which mirrors the results in the present study.

Preference for the LL option decreased as the value of the delay increased, which is consistent with several studies that evaluated the performance of SHR and WKY rats in delay-discounting tasks (Fox et al., 2008; Aparicio et al., 2019). Nevertheless, the discounting rate in our experiment is more progressive and reached a lower percentage of responses to the LL option than previously reported, probably due to an effect of increasing the delay by only 3 s per session, instead of increasing it to the double of the previous delay (Adriani et al., 2003; Anderson and Woolverton, 2005; Fox et al., 2008; Hand et al., 2009; Moreno et al., 2010; Íbias and Pellón, 2011; Aparicio et al., 2019). These results support the relationship between the length of the delay to the reinforcer and its subjective value.

Differences in the discounting rate between SHR and WKY rats have been reported in previous studies (Bizot et al., 2007; Fox et al., 2008; Hand et al., 2009; Sutherland et al., 2009; Íbias and Pellón, 2011, 2014; Wooters and Bardo, 2011; Orduña, 2015; Orduña and Mercado, 2017; Aparicio et al., 2019). In our experiment, no strain differences were observed, although visual inspection shows a trend for SHRs to discount more steeply than WKYs between delays of 9 and 18 s in Condition B.

Perhaps the possibility to engage in schedule-induced behavior prevented the expected results of steeper delay discounting in SHR in comparison to WKY, which resumed to a certain extent (but not to statistical significance) when there was no possibility to engage in schedule-induced behavior. During Condition B, in comparison to Condition A, SHRs tended to be more impulsive while WKYs tended to be more self-controlled. These tendencies did not reach statistical significance perhaps due to the previous experience on delay discounting without the possibility to engage in schedule-induced behavior, and/or because each delay value lasted only one session, or the small sample size of the groups (though similar to previous reports: e.g., Fox et al., 2008; Orduña and Mercado, 2017).

**Figure 6:**
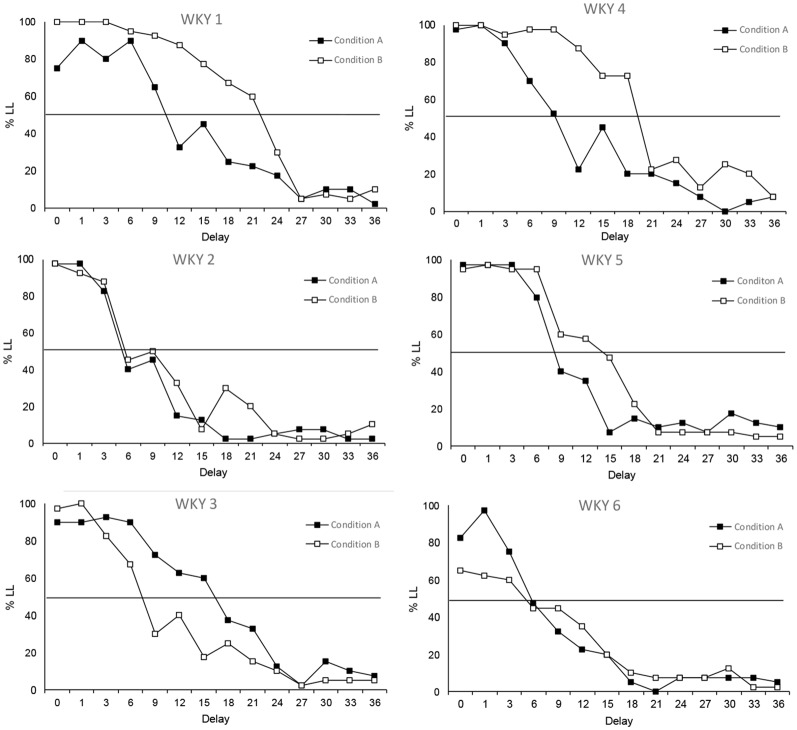
Individual percentage of responses of WKY rats to the LL option in each delay. Black circles depict data from Condition A and white squares from Condition B.

Schedule-induced behaviors do not seem to have much of an effect on the performance of SHR animals, which could be seen as a reflection of their normal excessive behavioral base rate (Sagvolden, 2000; Adriani et al., 2003; Fox et al., 2008; Hand et al., 2009; Aparicio et al., 2019). However, the tendency of WKY rats to behave more impulsively when they were able to engage in schedule-induced behavior is seemingly notorious. Figure 6 shows that 50% of the WKY rats displayed clearly steeper delay-discounting functions under Condition A in comparison to Condition B (WKY 1, WKY 4 and WKY 5), in contrast to the opposite result observed just in one rat (WKY 3). WKY rats do not usually show excessive behavior (Sagvolden, 2000; Adriani et al., 2003; Fox et al., 2008; Hand et al., 2009; Aparicio et al., 2019), but the excessiveness provided by the opportunity to engage in a schedule-induced behavior seemed to increase their activity during the task, making them behave like SHR subjects.

Differences between strains were observed in the development of schedule-induced behaviors. SHR developed schedule-induced drinking faster and at a higher rate than WKY rats, similar to what has been found in previous studies (Íbias and Pellón, 2011, 2014). Even though rats had simultaneous access to a bottle of water and a running wheel, only schedule-induced drinking was developed. This might be due to competition between licking and running, in which drinking was probably favored by the length of the inter-reinforcer interval used in this experiment (Roper, 1978; Pellón and Killeen, 2015), as it is one of the parameters that determine which schedule-induced behaviors are more likely to develop (Roper, 1978). Differences in schedule-induced drinking rates can be another reason to separate out SHR and WKY strains (see Moreno et al., 2010).

Distribution of licks during the response-reinforcer delay and the ITI exhibits the characteristic inverted U-shape function normally observed for schedule-induced drinking (e.g., López-Crespo et al., 2004; Íbias and Pellón, 2011; Álvarez et al., 2016). Since the delay initiated by the response becomes longer than the ITI, the inverted U-shape form is more defined in that time interval (Íbias and Pellón, 2011). Peak of the distribution of licks is usually located at the beginning of the interval, occurring during the first 15–20 s in the case of a regular presentation of food pellets (e.g., López-Crespo et al., 2004). In our study, licks during the response-reinforcer delay occurred during the first 3–9 s. This earlier location could be due to the short duration of the delays on the first delay-discounting sessions and the short experience with each delay value (only one session per delay) that could make it difficult for the rats to adjust to the interval length.

One of the arguments against schedule-induced behavior being considered operant is the early temporal location towards immediately after (rather than before) delivery of the reinforcer (Falk, 1971; López-Crespo et al., 2004; for an account of this, see Killeen and Pellón, 2013). In the present study, however, when rats had the opportunity to drink both before and after food reinforcement (LL trials), they allocated licks only during pre-food (the response-reinforcer delay) and not post-food (the ITI) periods, developing licking during the ITI just when there was no response-reinforcer delay (SS trials). These data can be interpreted as if pressing the lever and licking from the spout were part of the same behavioral pattern maintained by intermittent food reinforcement (Ruiz et al., 2016), being reinforcer effective given the systematic presence of behaviors in the context of spread reinforcement in time (Killeen and Pellón, 2013; Álvarez et al., 2016).

Impulsivity can be observed in two forms: cognitive impulsivity, determined by the choice, and motor impulsivity, understood as excessive behavior (Chudasama et al., 2003; Winstanley et al., 2004). Schedule-induced behaviors have been regarded as signs of motor impulsivity, but Íbias and Pellón (2011) proposed that schedule-induced behaviors could also reflect cognitive impulsivity because the amount of schedule-induced drinking relates to parameters of the upcoming reinforcer (López-Crespo et al., 2004), making schedule-induced behaviors indistinguishable from operant behaviors (Killeen and Pellón, 2013).

Nevertheless, developing schedule-induced behaviors during Condition A made rats exhibit excessive behavior. Our results seem to indicate that there is no difference between cognitive and motor impulsivity. This is in line with the view that schedule-induced behaviors are operants (Killeen and Pellón, 2013), but goes against the categorization of impulsivity in two types (cognitive and motor) because it does not seem to be a difference between them in the present study. Schedule-induced drinking could be part of the same behavioral pattern that determines the choice of subjects, as reported by other authors (Cleaveland et al., 2003; Machado and Keen, 2003; López-Tolsa and Pellón, in preparation). Similar findings have been observed in DRL schedules (Segal and Holloway, 1963) and the peak procedure (Mattel and Portugal, 2007), tasks that both involve self-control. Thus, impulsivity, in general, could be understood as excessive activity—hyperactivity (see also Íbias and Pellón, 2014).

Tendencies showed by each strain in this experiment point to be similar to previous findings reported with humans. Housden et al. (2010) compared Parkinson’s disease patients that had or did not have comorbid impulsive-compulsive spectrum behaviors. They found that patients with comorbid impulsive-compulsive spectrum behaviors showed highly elevated delay discounting. Furthermore, Pinto et al. (2014) compared OCD patients with participants with an obsessive personality, although both are marked by compulsions, they seem to differ in impulsivity, as they found that OCD patients discounted faster in intertemporal choice procedures. Finally, Sohn et al. (2014) found that OCD patients showed more impulsivity than healthy people in different tasks.

Schedule-induced drinking has been proposed as a model of compulsivity (Moreno and Flores, 2012) because they share features like excessiveness, persistence and having no obvious relation to the overall goal (reinforcement). Considering the results described above with humans and the tendencies seen in our experiment, we can start suggesting, but not stating, that schedule-induced behavior could serve as a model of compulsivity, in the sense that performance of SHR could make them comparable to patients with Parkinson’s disease with comorbid compulsivity and patients with OCD, while the tendency of WKY rats resembled participants in control groups of those studies (patients with Parkinson’s disease without comorbid compulsivity, patients with obsessive personality, and healthy people), but only in Condition B. By allowing rats to engage in schedule-induced behaviors in Condition A (which models compulsive behavior), we favored the occurrence of compulsive behavior in both strains of rats. This conclusion, however, requires further experimental confirmation given the limitation of the current statistical results and the previous experience of the subjects.

In conclusion, our results failed to document any effect of schedule-induced behavior on delay discounting, suggesting that schedule-induced behaviors do not reduce impulsivity. Nevertheless, a tendency can be observed that seems to support the idea of schedule-induced behavior as a model of compulsivity. No strain statistical differences were found, which suggest that the SHR strain is unsuitable as a model for compulsivity. Furthermore, the distribution and location of licks within time intervals of the delay-discounting task support the notion of schedule-induced behavior as operant.

## Ethics Statement

All care and experimental procedures were in accordance with the Spanish Royal Decree 53/2013 regarding the protection of experimental animals and with the European Union Council Directive 2010/63. UNED bioethics committee approved the experimental protocol.

## Author Contributions

SR designed research, performed the experiment, performed statistical analyses and wrote the initial version of the manuscript. GL-T designed research, assisted in programming the task, assisted in data analyses, contributed with writing. ES designed research, programmed the task, reviewed the writing. RP designed research, supervised work, reviewed the writing.

## Conflict of Interest

The authors declare that the research was conducted in the absence of any commercial or financial relationships that could be construed as a potential conflict of interest.
